# High prevalence of low-concentration antimicrobial residues in commercial fish: A public health concern in Bangladesh

**DOI:** 10.1371/journal.pone.0324263

**Published:** 2025-05-27

**Authors:** Md. Raihan Khan Nayem, Md. Rahim Badsha, Md. Kaisar Rahman, Shahneaz Ali Khan, Md Mazharul Islam, Md. Latiful Bari, John I. Alawneh, Ricardo J. Soares Magalhaes, Mohammad Mahmudul Hassan

**Affiliations:** 1 Remount Veterinary and Farm Corps, Bangladesh Army, Dhaka, Bangladesh; 2 Faculty of Veterinary Medicine, Chattogram Veterinary and Animal Sciences University, Chattogram, Bangladesh; 3 Al Arkkan Training Center, Dammam, Saudi Arabia; 4 School of Veterinary Medicine, Texas Tech University, Lubbock, Texas, United States; 5 Department of Animal Resources, Ministry of Municipality, Doha, Qatar; 6 Center for Advanced Research in Sciences, University of Dhaka, Dhaka, Bangladesh; 7 Plant Biosecurity and Product Integrity, Biosecurity Queensland, Department of Primary Industries, Brisbane, Australia; 8 Queensland Alliance for One Health Sciences, School of Veterinary Science, The University of Queensland, Gatton, Queensland, Australia; Bangladesh Agricultural University, BANGLADESH

## Abstract

Antibiotics are widely used in commercial fish farms in Bangladesh for therapeutic and prophylactic purpose, raising concerns about antimicrobial resistance (AMR) and environmental contamination. This study used Thin Layer Chromatography to detect antimicrobial residues in four commercially available fish species- Tilapia (*Oreochromis aureus*), Stinging catfish (*Heteropneustes fossilis*), Climbing perch (*Anabas testudineus*), and Pabda (*Ompok pabda*)—with 100 samples per species. Ultra High-Performance Liquid Chromatography quantified residues in a subset of 25 samples per species. The prevalence of Ciprofloxacin, Oxytetracycline, and Chlortetracycline residues varied significantly among fish species, with the highest prevalence observed for Ciprofloxacin in Tilapia (42%), Oxytetracycline in Pabda (41%), and Chlortetracycline in Tilapia (49%). Additionally, the prevalence of Levofloxacin and Chlortetracycline differed by sampling location, with the highest levels found in Jhawtala market, 27.5% for Levofloxacin and 53.8% for Chlortetracycline. Furthermore, residue concentrations were highest for Enrofloxacin in Climbing perch (69.32 µg/Kg) and Oxytetracycline in Pabda (88.73 µg/Kg). The highest Hazard Quotient (HQ) was for Enrofloxacin in Climbing perch (0.480), followed by Pabda (0.460), Stinging catfish (0.420), and Tilapia (0.387). While the HQ values were below 1.0, indicating no immediate toxicological risk, residues raise public health concerns due to the chance of potential AMR development. Further research is needed on antimicrobial bioaccumulation, indirect exposure sources, environmental contamination, and antimicrobial resistance in aquaculture and wild fish.

## Introduction

Bangladesh is one of the top fish-producing countries in the world [1], where fish accounts for approximately 60% of the total animal protein for human consumption. Aquaculture is a vital sector within Bangladesh’s agricultural industry, significantly contributing to the national economy [2,3]. However, infectious diseases, particularly bacterial infections, pose a major challenge to aquaculture in Bangladesh [4], leading to substantial economic losses for fish farms.

The use of antimicrobials is common in fish farming, especially in commercial aquaculture, to control bacterial diseases. In addition to medicinal use, antimicrobials are employed as growth promoters in commercial fisheries in many parts of the world [5,6]. Moreover, antimicrobials can also enter aquaculture systems through poultry litter when it is used as fish feed. In poultry farming, residues from antimicrobial treatments—used for disease prevention, treatment, or growth promotion—can be excreted and accumulate in litter, which may later contaminate aquaculture environments. However, the use of antimicrobials in animal and fish feed is officially prohibited in Bangladesh [7]. Despite this, there is no established antimicrobial stewardship practice in the country and reliable data on antimicrobial usage in livestock and aquaculture remain scarce [8].

Antimicrobial residues (ARs) are pharmacologically active components or their metabolites that remain in the foodstuffs obtained from animals treated with these drugs [6,9]. Antimicrobial residues can occur in commercial fish when the drugs are administered in higher doses or without recommendations from a registered veterinarian [10,11]. To reduce the hazards related to AR, withholding periods (WHP) and maximum residue limit (MRL) of an antimicrobial has been suggested by the Food and Agricultural Organization (FAO) of the United Nations to determine the safety of animal-originated food sources [12]. While the WHP is the interval between the last dose of veterinary medication given to an animal and the subsequent slaughtering of the animal or use of its products, such as meat, milk, or egg, for human consumption, the ‘MRL is the maximum residue concentration from a veterinary drug’ permitted quantities of drugs or metabolites [13] in foods originating from animals that are safe for consumers [14].

A low level of antimicrobial exposure in the Environment can lead to high-level resistance [15]. The lack of attention to the WHP and MRL can pose significant health risks associated with the presence of ARs [9]. Such as toxicological effects and also increasing the probability of developing antimicrobial resistance (AMR) against the commonly used antimicrobials, such as ciprofloxacin and oxytetracycline [16]. Although steps have been taken to maintain the MRLs worldwide, MRLs differ from place to place. Even within a single country, MRLs in animal products may vary depending on local food safety regulatory agencies and drug usage patterns. Most developing countries have not yet developed their MRLs [17]. Acceptable daily intake (ADI) estimates the amount of a veterinary drug on a body weight basis [14] consumed daily over a lifetime without any health risk to the consumer. ADI is a critical standard from toxicological studies based on the No Observable Effect Level (NOEL) and safety factors [18].

A previous study found that approximately 21% of the fish farms in Bangladesh had used at least one antibiotic within the last 14 days, often based on recommendations from fish feed dealers or drug sellers [19]. When WDP, MRL, and ADI guidelines are not followed, there is a heightened risk of developing resistance to commonly used antimicrobials. These ARs can also be transferred to humans through the food chain via fish consumption [16]. However, due to the limited research, the full extent of this risk remains difficult to evaluate. The current study aims to assess the presence and concentration of antimicrobials in commonly consumed fish species in Bangladesh and evaluate the potential risks these residues may pose to consumers.

## Materials and methods

### Ethical approval

This study was conducted by following the Declaration of Helsinki, and the protocol was approved by the Ethics Committee of the Chattogram Veterinary and Animal Sciences University, Bangladesh (permit reference number: CVASU/Dir (R and E) EC/2019/126 (02), Date: 29 December 2019). Participants, who were fish sellers in wet markets, provided informed consent after the study’s purpose, procedures, and their rights were clearly explained in their local language. Verbal consent was obtained due to cultural norms and literacy considerations, with approval from the relevant ethics committee. Each verbal consent was documented in a consent log and witnessed by an independent third party who confirmed the participant’s understanding and voluntary agreement.

### Study area and sampling

The study was conducted on four commercially cultivated fish species: Tilapia (*Oreochromis aureus*), Stinging catfish (*Heteropneustes fossilis*), Climbing perch (*Anabas testudineus*), and Pabda (*Ompok pabda*). Samples were collected from various wet markets in the Chattogram Metropolitan Area, Chattogram district, Bangladesh, from October 2020 to March 2021. To minimize sampling bias, simple random sampling was employed at all stages of sampling. Five wet markets (Bahaddarhat, Chawkbazar, Jhawtala, Pahartali, and Reazuddin Bazar) were randomly selected from a total of 40 markets. At each selected market, five vendors were randomly chosen, and four fish samples were collected from each vendor, resulting in 80 samples per market and a total of 400 fish samples overall.

### Thin layer chromatography

#### Sample processing and preparation.

After washing with tap water, the flesh from each fish was separated from the bones individually and pasted. Then, four grams of pests were added with 10 ml phosphate-buffered saline, and two ml of 30% trichloroacetic acid were vortexed. The mixture was centrifuged at 3500 rpm for 20 minutes, and five milliliters of the supernatant were collected and filtered. This was then mixed with diethyl ether by vortexing and left to settle down to separate the fatty portion of the sample. The extracted sample was then collected with a dropper to a 5 ml cryovial [20].

#### Preparation of standard and mobile phase.

Standards of Ciprofloxacin, Enrofloxacin, Doxycycline, Levofloxacin, Oxytetracycline, and Chlortetracycline were collected from commercial sources. However, if the product was in powder form, then 0.1 g of the powder was mixed with 4 ml Methanol. In addition, a mobile phase or solvent system was prepared by mixing 50 ml of methanol with 50 ml of acetone in the TLC tank to perform TLC.

#### Pointing and running of TLC.

The TLC plate was cut to fit the size of the TLC tank. A line was drawn above the solvent level, and three spots were made on the line, spaced about 2 cm apart. The first spot was used for the standard, and the other two were used for the samples. The plate was then dried for a minute.

The prepared TLC plate was placed in a TLC tank containing the mobile phase. It was left for about 15 minutes until the solvent rose to the top of the TLC plate. The plate was then removed and dried. The dried TLC plate was placed in a UV chamber and examined under ultraviolet light at 256 nm. The distance travelled by the standard and sample spots from the start line was measured in centimeters. The outline of the top spot was marked with a pencil [20].

#### Determination of retardation factor.

The retardation factor (Rf) was determined to define the relative migration rate of substances under various conditions. Rf is the ratio of the distance moved by the substance to the distance moved by the solvent. To calculate the Rf values, the distance each spot travelled from the start line was measured in centimeters [20]. The relative migration rates of substances were used to identify unknown chemicals by comparing them to known standards.

#### Ultra high-performance liquid chromatography.

A total of 100 samples (25 from each fish type) were chosen randomly from the TLC-positive samples for Ultra-High-Performance Liquid Chromatography (UHPLC) to determine the concentration of antimicrobial residues.

#### Sample preparation.

Two grams of groundfish flesh were placed into a Falcon tube. Then, 8 ml of 30% trichloroacetic acid was added and thoroughly mixed by vortexing. The mixture was centrifuged at 3000 rpm for 20 minutes. A syringe filter was used to filter the supernatant, which was then collected into a vial.

#### Preparation of standards.

To prepare standard solutions, 10 mg of each of Ciprofloxacin, Oxytetracycline, Levofloxacin, and Enrofloxacin were weighed. The detection limits (LOD) and quantification limits (LOQ) for each compound were as follows: Ciprofloxacin (LOD: 0.05–0.5 ng/mL and LOQ: 0.1–1.5 ng/mL), Oxytetracycline (LOD: 0.5–2.0 ng/mL and LOQ: 1.5–6.0 ng/mL), Levofloxacin (LOD: 0.05–0.5 ng/mL and LOQ: 0.1–1.5 ng/mL), and Enrofloxacin (LOD: 0.05–0.5 ng/mL and LOQ: 0.1–1.5 ng/mL). Each weighed antimicrobial was dissolved in 10 ml of 5% trichloroacetic acid in a Falcon tube to reach a stock concentration of 1000 ppm. The solutions were vortexed for 5 minutes and filtered through a 0.45 μm nylon filter. Serial dilutions were performed to obtain concentrations ranging from 1250 ppb to 15,000 ppb (i.e., 1.25–15.00 μg/ml) using the trichloroacetic acid solution. The supernatant was again filtered through a 0.45 μm nylon filter prior to infection into the UHPLC [21].

#### Performing the test.

UHPLC was performed on a SIL 20 series Prominence UHPLC system (Shimadzu, Japan), equipped with an autosampler (Model SIL-20 AC), dual pumps (Model 20 AD), a column oven (Model CTO-20A), a vacuum degasser (Model DGU-20A), and a UV-visible detector (Model SPD-20A). Data acquisition and analysis were performed using LC solution software. An analytical reversed-phase C-18 (Luna 5μ, 250 x 4.6 mm, Phenomenex, Inc., Japan) was used [21].

The mobile phase consisted of acetic acid (10%) and acetonitrile (90: 10). UV detection was set at 280 nm. The total run time was 15 minutes, with a flow rate of 1.0 mL/min. The column temperature was maintained at room temperature, and the injection volume was 20 μL. Elution was performed under Isocratic conditions.

Sample-containing vials were placed in the input section of the UHPLC machine for auto-sampling. After completion of the UHPLC run, chromatographic peaks were obtained as output. These peaks were calibrated using the software to determine the residue concentrations in the samples by comparing them with chromatographic peaks of standard solutions. For our calculations, non-detectable residues were considered to be zero. The MRLs established by national and international organizations for the studied antibiotics are provided in Table 1.

**Table 1:** Antimicrobial concentrations at national and international level, used as standard reference value in this study.

Antibiotics	Bangladesh MRLs (µg/kg)	EU MRLs (µg/kg)	FAO/WHO MRLs (µg/kg)	References
Ciprofloxacin	Not specified	100	Not specified	[22,23]
Enrofloxacin	Not specified	100	Not specified	[22,23]
Levofloxacin	Not specified	Not specified	Not specified	[22,23]
Tetracycline	Not specified	100	200	[22,23]

### Statistical analysis

All the TLC and UHPLC results data were organized in Microsoft Excel 2013 and sorted according to fish type and wet market. Further analysis was performed using R software (R Studio, version 1.4.1717). Descriptive statistics were performed to identify the TLC and UHPLC positive samples. Univariate analyses were performed for specific antimicrobials tested in TLC to obtain the prevalence and 95% confidence interval. For the TLC method, the prevalence of ARs for sample type and location were analyzed using the Chi-square test. Logistic regression was performed to examine whether the prevalence of ARs significantly differed among fish species. Additionally, the concentration of the antimicrobials in UHPLC were tested for normality using Shapiro–Wilk test, then the ARs concentrations were tested for sample type and location using Kruskal-Wallis test. A p-value less than 0.05 was considered statistically significant.

We used the Hazard Quotient (HQ) model to assess the toxicological risk of consuming fish containing antibiotic residues. The HQ estimation includes the Acceptable Daily Intake (ADI) as a denominator, defined as an estimated amount of residue that can be ingested daily over a lifetime without any appreciable health risk, expressed on a bodyweight basis. ADI value of Oxytetracycline is 30 µg/kg/day [24], and Quinolones (Ciprofloxacin, Enrofloxacin, and Levofloxacin) are 0.15 µg/kg/day [25]. The numerator of the HQ is the Estimated Daily Intake (EDI), which is considered the mean antibiotic residue concentration value, and the average daily fish consumption based on a 60 kg body weight was considered. Per capita, fish consumption in Bangladesh is approximately 62.58 g/day [26]. The following equations were used for the EDI and HQ estimation [27]:


$$\begin{array}{c} \text{EDI}=\frac{\left(\text{concentration\textbackslash{} of\textbackslash{} residue\textbackslash{} as}\mu \text{g/kg})\text{x}(\text{daily\textbackslash{} intake\textbackslash{} of\textbackslash{} food\textbackslash{} in\textbackslash{} kg/person}\right)}{\text{Adult\textbackslash{} body\textbackslash{} weight}(60\text{kg})} \end{array}$$

The HQ is the ratio of the potential exposure to a substance and the level at which no adverse effects are expected [27].


$$\begin{array}{c} \text{HQ}=\frac{\text{Estimated\textbackslash{} daily\textbackslash{} intake}(\text{EDI})}{\text{Accepted\textbackslash{} daily\textbackslash{} intake}(\text{ADI})} \end{array}$$

An HQ less than or equal to one indicates negligible hazard, while an HQ more fantastic than one suggests toxicological effects on the health of consumers [27].

## Results

### Prevalence of antimicrobial residues

The overall prevalence of ARs in the tested fish samples by TLC were 25% (95%CI: 20.89–29.60) (S1 Table). Species-wise analysis showed that Tilapia had the highest prevalence at 86%. (95% CI: 77.62–92.12), followed by Pabda at 77% (95% CI: 67.51–84.82), Stinging catfish at 75% (95% CI: 65.34–83.12) and Climbing perch at 62% (95% CI: 51.74–71.52) (Table 1). The chi-squared test showed a statistically significant difference of ARs prevalence by fish species. Logistic regression analysis revealed that, compared to Catfish, Koi had a significantly lower prevalence of ARs detected by TLC (p < 0.05), Tilapia had a borderline significantly higher prevalence (p = 0.052), and Pabda showed no significant difference (p > 0.05) (Table 2). However, no significant differences in ARs prevalence were detected based on sampling sources in TLC.

**Table 2:** Prevalence of TLC-positive fish samples based on sample types and wet market.

Factor	Category	TLC		
		N	Positive, % (95%CI)	P value
**Sample type**	**Stinging catfish**	100	75, 75, 65.34-83.12	<0.01
	**Climbing perch**	100	62, 62, 51.74-71.52	
	**Pabda**	100	77, 77, 67.51-84.82	
	**Tilapia**	100	86, 86, 77.62-92.12	
**Wet market**	**Bahaddarhat**	80	54, 67.50, 56.1-77.55	0.27
	**Chawkbazar**	80	62, 77.50, 66.79-86.08	
	**Jhawtala**	80	65, 81.25, 70.96-89.11	
	**Pahartali**	80	62, 77.50, 66.79-86.08	
	**Reazuddin Bazar**	80	57, 71.25, 60.04-80.82	

When the TLC results of each antimicrobial were analyzed separately, Tilapia fish exhibited the highest prevalence of ciprofloxacin (42.0%) and chlortetracycline (49.0%) compared to other fish species. Pabda fish showed the highest prevalence of oxytetracycline (41.0%) and doxycycline (38.0%). Additionally, fish from Jhawtala had the highest prevalence of levofloxacin (27.0%%) and chlortetracycline (53.8%) among all the wet markets (Table 3).

**Table 3:** Prevalence of Ciprofloxacin residues in fish samples by TLC.

Factor	Category	N	Ciprofloxacin		Enrofloxacin		Levofloxacin		Oxytetracycline		Chlortetracycline		Doxycycline	
			Positive (%)	p	Positive (%)	p	Positive (%)	p	Positive (%)	p	Positive (%)	p	Positive (%)	p
Sample type	Stinging catfish	100	31 (31.0)	<0.001	22 (22.0)	0.124	17 (17.0)	0.126	27 (27.0)	0.017	35 (35.0)	<0.001	28 (28.0)	0.005
	Climbing perch	100	20 (20.0)		17 (17.0)		17 (17.0)		23 (23.0)		21 (21.0)		16 (16.0)	
	Pabda	100	10 (10.0)		31 (31.0)		27 (27.0)		41 (41.0)		43 (43.0)		38 (38.0)	
	Tilapia	100	42 (42.0)		22 (22.0)		15 (15.0)		24 (24.0)		49 (49.0)		32 (32.0)	
Wet market	Bahaddarhat	80	20 (25.0)	0.372	17 (21.3)	0.647	14 (17.5)	0.012	18 (22.5)	0.275	31 (38.8)	0.005	19 (23.8)	0.332
	Chawkbazar	80	19 (23.8)		18 (22.5)		21 (26.3)		30 (37.5)		28 (35.0)		18 (22.5)	
	Jhawtala	80	27 (33.8)		19 (23.8)		22 (27.5)		23 (28.8)		43 (53.8)		28 (35.0)	
	Pahartali	80	21 (26.3)		23 (28.8)		12 (15.0)		24 (30.0)		21 (26.3)		26 (32.50)	
	Reazuddin Bazar	80	16 (20.0)		15 (18.8)		7 8.8)		20 (25.0)		25 (31.3)		23 (28.8)	

### Concentration of antimicrobial residues

**Fig 1:**
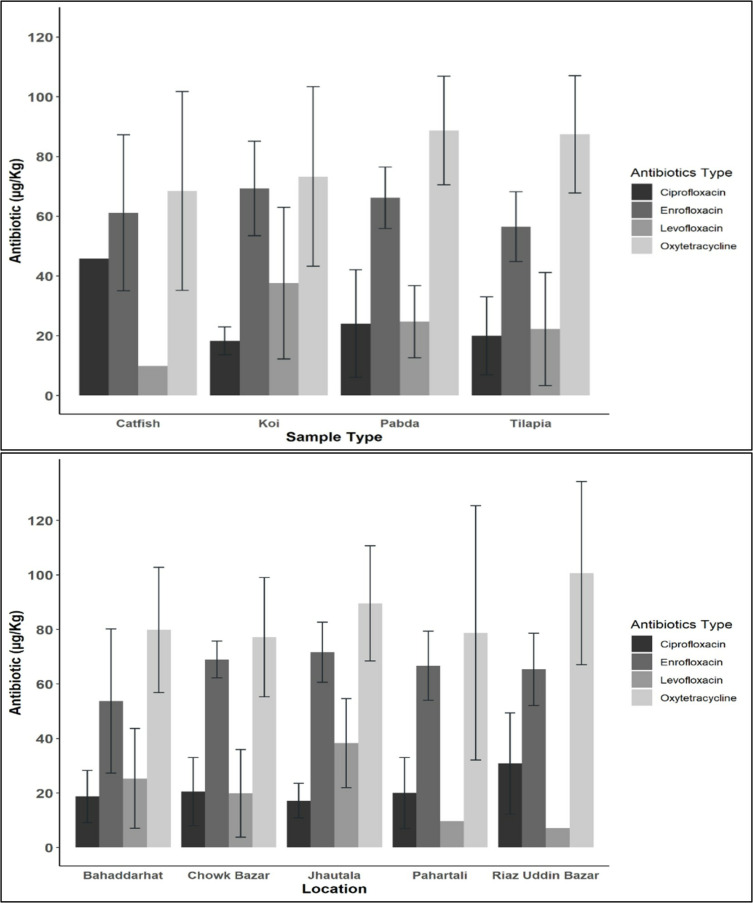
Concentration level of different antibiotics residues based on fish type (top panel: sample types; bottom: locations).

The average concentration of ARs in the fish samples were 73.22 µg/Kg (95%CI: 59.39–87.06) (S1 Table). The concentrations of the ARs varied significantly among fish species (p < 0.001), indicating species-specific differences in residue levels. Ciprofloxacin residues were found in the highest concentration (45.85 µg/Kg) in Catfish, while enrofloxacin (69.32 µg/Kg) and levofloxacin (37.59 µg/Kg) were most concentrated in Koi. Oxytetracycline was found in the highest concentration (88.73 µg/Kg) in Pabda (Fig 1). In terms of location, levofloxacin and oxytetracycline concentrations differed significantly across market locations (p < 0.001), while no significant variation was observed for ciprofloxacin and enrofloxacin by location. Oxytetracycline were detected at the highest concentration in fish from Riajuddin Bazar, whereas the highest levels of levofloxacin were found in fish from Jhawtala.

### Hazard quotient

Based on the mean value of antimicrobial residues in different fishes, Stinging catfish had the highest HQ value for ciprofloxacin (0.320). On the other hand, the climbing perch was found to have the highest HQ value for Enrofloxacin and Levofloxacin, with values of 0.48 and 0.26, respectively. However, oxytetracycline was detected to have the highest HQ value in Pabda fish (Table 4).

**Table 4:** Estimation of risk assessment by Hazard Quotient for mean concentration of residues in fishes.

Antimicrobial	Fish	EDI (µg/Kg/day)	ADI (µg/Kg/day)	Hazard Quotient
Ciprofloxacin	Stinging catfish	0.048	0.15	0.320
	Climbing perch	0.019	0.15	0.127
	Pabda	0.025	0.15	0.167
	Tilapia	0.021	0.15	0.140
Enrofloxacin	Stinging catfish	0.063	0.15	0.420
	Climbing perch	0.072	0.15	0.480
	Pabda	0.069	0.15	0.460
	Tilapia	0.058	0.15	0.387
Levofloxacin	Stinging catfish	0.010	0.15	0.067
	Climbing perch	0.039	0.15	0.260
	Pabda	0.026	0.15	0.173
	Tilapia	0.023	0.15	0.153
Oxytetracycline	Stinging catfish	0.071	30	0.0024
	Climbing perch	0.077	30	0.0026
	Pabda	0.093	30	0.0031
	Tilapia	0.091	30	0.0030

## Discussion

In Bangladesh, antimicrobials are frequently used as fish feed additives and for disease treatment in aquaculture [11,28], although it is not supported by law. This practice is concerning, as the country lacks antimicrobial stewardship, increasing the risk of ARs in fish and contributing to the AMR. Although several studies have investigated AMR in fish in Bangladesh [29–32]. This study is the first to quantify the HQ of antimicrobials in fish within this country. A similar study was conducted in Saudi Arabia showed that the estimated daily intake of fluoroquinolones, sulfonamides and macrolides were ranging from 0.10 to 6.61 ng/kg/ BW/day, which was considered not a serious risk to consumers [33]. However, the most commonly used antibiotics in the aquaculture industries in Bangladesh include Ciprofloxacin, Enrofloxacin, Levofloxacin, Oxytetracycline, Doxycycline, Colistin sulphate, and Neomycin [34]. A 2015 study found Amoxicillin and Oxytetracycline residues in Climbing Perch, Tilapia, Rui, Bombay duck, and Shrimp [31], with Oxytetracycline residues present in 8.3% of Climbing perch and 3.03% of Tilapia samples [31], figures considered low compared to this study. In contrast, this study detected Ciprofloxacin, Enrofloxacin, Levofloxacin, Oxytetracycline, Chlortetracycline, and Doxycycline residues with high prevalence and significant concentrations. In 2016, a survey conducted in the Sylhet district found Oxytetracycline residues in Tilapia at a mean concentration of 38.88 µg/Kg [30], which was lower than the comparisons found in this study. Another 2016 study in Sylhet determined Oxytetracycline residues in Pungas at a mean concentration of 35.11 µg/Kg [32]. Additionally, a 2013 study in Chattogram detected high concentrations of Chloramphenicol residues in Climbing perch, Tilapia, Rui, Pungas, and Trout [29]. In contrast, this study detected Ciprofloxacin, Enrofloxacin, Levofloxacin, Oxytetracycline, Chlortetracycline, and Doxycycline residues with high prevalence and significant concentrations.

The HQ indicates the potential risk to human health from consuming fish with these residues, highlighting the potentially hazardous effects [27]. Using the calculated mean concentrations of antimicrobial residues, HQs were determined for various antimicrobials in fish. The highest HQ was found for Enrofloxacin in Climbing Perch, followed by Pabda, Stinging Catfish, and Tilapia, suggesting potential health risks. Ciprofloxacin residues also presented a significant HQ. In particular, HQs for Ciprofloxacin and Levofloxacin residues in Stinging catfish, Pabda, Tilapia, and Climbing Perch were notable. In contrast, Oxytetracycline residues in Pabda, Tilapia, Climbing Perch, and Stinging catfish were associated with negligible HQs.

Although the HQs for Ciprofloxacin, Enrofloxacin, Levofloxacin, and Oxytetracycline residues were below 1.0, suggesting no significant toxicological effects on consumers’ health based on average fish consumption. However, higher fish consumption could pose toxicological risks if the EDI exceeds the ADI, particularly for Enrofloxacin, Ciprofloxacin, and Levofloxacin residues. While immediate toxic effects may not be evident, the presence of these residues raises concerns about their long-term impact, particularly in the context of AMR. We detected a low level of antibiotic residues in the present research compared to the data from the other studies in Bangladesh [30–32]. Low-level antibiotic exposure can drive genetic mutations in microbes, as evidenced by a study showing that one month of exposure to oxytetracycline (5µg/L) in Zebrafish significantly increased the abundance of *Pseudomonas aeruginosa* and the resistant gene *tet*E [35]*.* AMR in human pathogens is now a critical global public health issue [36], with mounting evidence linking antimicrobial use in food-producing animals and resistance in human infections [37].

Inadequate treatment records, poor management, failure to observe drug withdrawal periods, and the accessibility of antimicrobials to commercial fish farm owners are the leading causes of residues in commercial fish. Additionally, consumers are often unaware of the health risks of consuming these residues. National regulatory bodies should take appropriate steps to implement strict legislation on antimicrobial use and raise public awareness. The use of antimicrobials as feed additives, along with the use of poultry offal and litter in aquaculture, may result in low-level antimicrobial exposure in fish [38]. Antimicrobial residues in fish are ultimately due to poultry and livestock waste in Bangladeshi aquaculture. It is concluded that the probable public health hazard of AMR arises from consuming sub-therapeutic levels of antimicrobials from food-producing animals [39]. Several reports have described the relationship between animal antimicrobial use and the development of resistance to human pathogens. If any antimicrobial residue exceeds the MRL, it is a severe public health concern [40]. Consumers should be made aware of this issue, and proper food processing techniques might help reduce residue levels [31]. Surveillance data indicated a growing trend of antimicrobial resistance in livestock across Bangladesh [41], highlighting the critical need for enhanced monitoring, stricter antimicrobial stewardship, and integrated One Health strategies to mitigate the risk of transmission to humans and the environment. Implementing antimicrobial stewardship in food animal production is crucial to reducing resistance and preserving vital drugs, especially drugs under the quinolone and tetracycline groups, for both human and veterinary medicine.

## Conclusion

Several samples contained antimicrobial residues, some at notably high concentrations, highlighting concerns about improper drug withdrawal practices in commercial fish farms. The misuse of antimicrobials in aquaculture has contributed to the emergence of resistant pathogens and resistance genes, posing risks to fish production and public health. Antimicrobial-resistant bacteria in fish underscores the need for responsible antimicrobial use. Further research is needed on antimicrobial bioaccumulation, alternative sources of antimicrobial exposure in fish beyond direct treatment, environmental contamination from aquaculture affecting both farmed and wild fish populations, and the presence of resistant bacteria and resistance genes.

## Supporting information

S1 Table Raw data of TLC and UHPLC. (XLSX)
